# Socio-demographic and psychiatric profile of patients hospitalized due to self-poisoning with suicidal intention

**DOI:** 10.1186/s12991-022-00393-3

**Published:** 2022-06-09

**Authors:** Maja Lumpe, Johannes Schurr, Christian Rabe, Armin Ott, Tobias Zellner, Michael Rentrop, Florian Eyer, Stefanie Geith

**Affiliations:** 1 Division of Clinical Toxicology and Poison Control Centre Munich, Department of Internal Medicine II, School of Medicine, Technical University of Munich, Munich, Germany; 2 Lungenfachkliniken München-Gauting, Gauting, Germany; 3 Staburo GmbH, Munich, Germany; 4 Department of Psychiatry and Psychotherapy, Klinikum Rechts der Isar, Technical University of Munich, Munich, Germany; 5 kbo-Inn-Salzach Clinic, Wasserburg am Inn, Germany

**Keywords:** Suicide, Suicide attempts, Poisoning, Psychiatric disorder

## Abstract

**Objective:**

To identify the psychiatric profile of patients hospitalized due to self-intoxication associated with suicide-related behavior (SRB).

**Methods:**

In this retrospective single-center study, records of consecutive patients treated for suicidal poisoning in our Clinical Toxicology unit between 1st January 2012 and 31st December 2016, who received at least one psychiatric exploration during their inpatient stay, were analyzed with regard to epidemiological data, ingested substances, psychiatric and somatic comorbidities, suicidal circumstances and follow-up therapy.

**Results:**

Out of 1289 hospitalized patients, 1090 patients with complete data were analyzed. Mean age was 40.5 ± 17.2 years, 66.7% were female. 32.0% of patients had previously engaged in SRB, in 76.3% intention was suicidal. 64.7% of patients had a pre-existing psychiatric disorder (PD). Patients with a pre-existing PD more often displayed prior SRB than those without PD (40.7% vs 15.3%; *p* < 0.001; Fisher′s exact test), used long-term/on demand medication (70.2% vs 38.9%; *p* < 0.001), distanced themselves from the current suicide attempt (65.9% vs 50.8%; *p* < 0.001) and had no detectable trigger (38.7% vs 18.1%; *p* < 0.001). Partnership conflict was the most commonly named trigger, and it was documented more often in patients without than in those with PD (41.6% vs 25.6%). After psychiatric reevaluation, most patients were diagnosed with mood disorders (29.7%) and stress disorders (17.0%); 32.8% of patients had a combination of two or more PDs.

**Conclusion:**

Hospitalization due to self-poisoning is associated with pre-existing PD, prior SRB and access to psychiatric medication. Detection of these risk factors could allow timely introduction of effective preventive measures tailored to particularly vulnerable subgroups and appropriate relief. However, lack of a detectable trigger in many cases may hamper the identification of those at risk.

**Supplementary Information:**

The online version contains supplementary material available at 10.1186/s12991-022-00393-3.

## Introduction

Around 800,000 people worldwide die from suicide every year, with suicide being the second most common cause of death among 15–29 years old [1]. Although the number of suicides in Germany has almost halved since 1980, the number of suicides in the last decade has remained at a relatively constant level of just under 10,000 deaths per year [2] and thus represents a relevant socio-economic and medical problem of interest to the entire community. Nevertheless, these numbers represent only the completed suicides. Suicide attempts are up to 10–40 times more common than completed suicides [3]. Given the comparatively low mortality rate of suicidal intoxications [4–6] and underreporting, an actual incidence is most likely considerably higher [7, 8].

One of the strongest indicators of suicidal behavior or death by suicide is a previous suicide attempt [9–14]. For instance, prior suicide attempt is more strongly associated with suicidal behavior than presence of substance-related disorder, mood disorders, adverse marital status or adverse employment status [10]. Furthermore, it was shown that previous suicide attempt is also an important predictor for deliberate self-poisoning using pesticides or gas [14]. Among the patients hospitalized due to deliberate self-poisoning, Suokas et al. found an up to 50 times higher suicide mortality rate during the 5 years from the first suicide attempt, compared to the normal population [15]. Another important risk for suicide is the presence of psychiatric disorder (PD). Previous studies identified patients with any type of PD at increased risk of suicide with the highest suicide rates in those suffering from psychotic, mood and personality disorders [16, 17]. Therefore, describing the identifying characteristics of patients at risk for suicide is essential for the development of actionable prevention strategies.

The aim of the present study was to analyze the psychiatric and socio-demographic profile of individuals at high risk of suicide-related behavior (SRB) involving deliberate self-poisoning to better understand subgroups at risk and to refine increasingly tailored prevention strategies.

We hypothesized that (i) the presence of a psychiatric illness or a specific psychiatric disorder is associated with suicidal poisoning; (ii) there are differences in terms of substance choice, substance source, suicide planning and trigger factors between patients with previously unremarkable psychiatric history and those with psychiatric disorder and between the different psychiatric disorders, and (iii) underlying psychiatric disorder and profile of used substance determines further treatment. We retrospectively analyzed the data of a large group of patients hospitalized due to suicidal self-poisoning. To this end, we employed an interdisciplinary approach involving examination of data obtained within psychiatric interviews, toxicological examinations, and clinical outcomes data.

## Methods

### Study design and setting

**Fig. 1:**
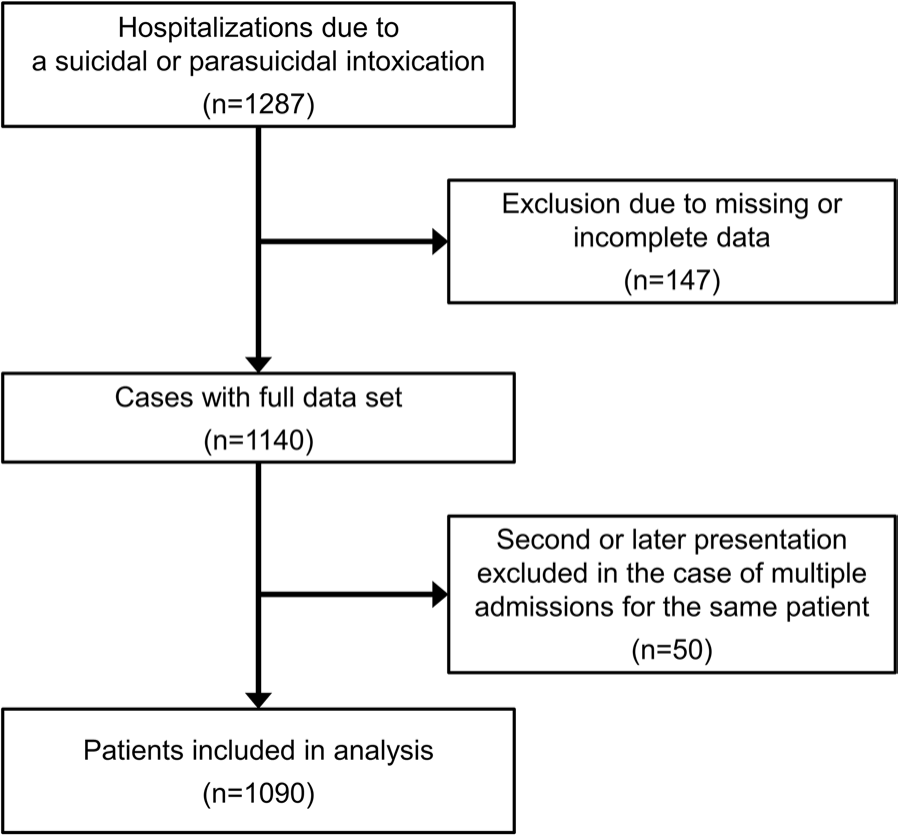
CONSORT diagram

This was a retrospective single-center study performed at the department of clinical toxicology in a tertiary university hospital in Bavaria, Germany. The department combines a poison control center, a toxicology laboratory, and a clinical unit consisting of an intensive care unit (ICU) and an intermediate care unit (IMC). Combination of ICU and IMC with a daily consultative co-care of PDs by psychiatrists enables a holistic treatment of patients tailored to the severity of the poisoning with a step-up and step-down of the treatment intensity. Between 1 January 2012 and 31 December 2016, 1287 patients were admitted to our department due to a suspected suicidal or parasuicidal intoxication (Fig. 1).

After excluding 147 patients due to missing or incomplete data and considering only the chronologically first presentation in the case of multiple admissions (*n* = 50, Fig. 1), 1090 patients were finally included in the analysis. Charts were reviewed in the context of SRB according to the classification proposed by Silverman et al. [14, 18] although several other definitions for intentional self-injury have been proposed in the literature, each with its own justification [19–21]. Patients were psychiatrically evaluated at least once during their hospitalization (except in the event of a fatal outcome) by a board-certified psychiatrist. Confirmation of the intentional self-harm as well as distinction between suicidal and parasuicidal intent was made within the psychiatric consultation. In cases where a patient has not been seen by a psychiatrist, the intent was determined at the discretion of the admitting physician considering the available data. In fatalities, intentional self-harm was assumed when a farewell letter had been found or relatives confirmed that a patient expressed the wish to die. The study was approved by the local Ethics Committee (No. 270/16 S), and it was conducted in compliance with the Declaration of Helsinki.

### Data collection

Patient data were extracted from the electronic medical records at the hospital and entered manually into a Microsoft Access database. For patients with more than one clinical admission, only the demographics of the first SRB was included in the analysis.

For age-grouped analyses, patients were classified into four groups: < 18 years, 18–44 years, 45–64 years, and > 64 years. The severity of clinical outcome was graded using the poisoning severity score (PSS, [22]) at admission, after 24 h and at further time points if necessary. For statistical analyses, we only considered the maximum score during the entire stay. Fatal outcome was defined as death due to either a direct toxic effect or an immediate complication (e.g., aspiration pneumonia).

Previous psychiatric medication, psychiatric treatment, and the pre-existence of PD was ascertained (“PD yes/no”). Up to three pre-existing PDs were grouped according to the ICD classification of mental and behavioral disorders as follows: disorder due to the use of psychoactive substance, referred to as addiction (F10-19); schizophrenia, schizotypal and delusional disorders, referred to as schizophrenia (F20-29); mood disorders (F30-F39); anxiety, dissociative, stress-related, somatoform and other nonpsychotic mental disorders, referred to as stress disorders (F40-49); behavioral syndromes associated with physiological disturbances and physical factors, referred to as personality disorders (F50-59); a combination of these groups or another PD (F00-09, F60-99) and no psychiatric disorder.

During the hospital stay, several patients were diagnosed with new or additional PD. Therefore, each patient was assigned a single PD diagnosed during the treatment at hospital, regardless of a pre-existing psychiatric illness. In cases where several or different pre-existing or newly diagnosed PD were documented, a “combined disorder” was assigned.

Further analyzed data included information on prior suicidal ideation, announcement of suicide (verbal or written), farewell letter, family burden regarding SRB, type of trigger and self-harm. The type and number of used substances were derived from the patient’s statement, outpatient services, companions, and toxicological analysis. The ingested substances were categorized into several pharmacologically defined groups. Ibuprofen, naproxen, diclofenac, acetylsalicylic acid, acetaminophen and metamizole were collectively termed as non-opioid analgesics while category z-drug included the generics zolpidem and zopiclone. Up to eight substances were recorded per patient, with each substance group only being recorded once per patient. Additionally, we evaluated whether patients distanced themselves from suicidal ideation and what type of follow-up therapy, if any, was applied after somatic stabilization.

### Statistical analysis

Data were analyzed descriptively using SPSS Statistics for Windows (version 25, IBM Corp., Armonk, NY, USA) and R (version 3.5.2; R Foundation for Statistical Computing, Vienna, Austria). Continuous variables are described as mean ± standard deviation (SD) and categorical variables are presented as absolute and relative frequencies. Nominal and ordinal variables were analyzed by Pearson’s Chi-squared test or Fisher’s exact test for sample sizes of five or fewer and interval scaled variables were assessed by Student's *t*-test. *p*-values ≤ 0.05 were considered statistically significant; because of the exploratory character of this study, we did not adjust for multiple testing.

## Results

### Patient characteristics

Tables 1 and 2 display patient characteristics by sex and age, respectively. 66.7% (*n* = 727) of patients were female and 55.3% (*n* = 603) of all patients were 18–44 years old. The mean age was 40.47 ± 17.24 years. On average, 1.90 ± 1.35 substances (median 1.00; min 1, max 13) were ingested per patient. In 68.3% (*n* = 744) of patients, this was the first instance of SRB, and in 31.7% (*n* = 346) of patients had at least one previous SRB. Patients had a mean history of 1.54 ± 2.83 SRB (median 1.00; min 1, max 86), with a statistically significant difference between males and females (*p* = 0.007). In 64.7% (*n* = 705) of patients a PD was known. Furthermore, in 55.0% (*n* = 600) of cases PD was newly diagnosed, (i.e., patients were diagnosed with PD for the first time, an additional PD has been diagnosed, or pre-existing diagnosis has been changed). The distribution of males and females differed in the terms of known PD, median number of prior SRB, intention, and follow-up therapy (Table 1).

**Table 1:** Patient characteristics according to sex

	Male *n* = 363 (33.3%)	Female *n* = 727 (66.7%)	Total *n* = 1090 (100.0%)	*p*-value
Known PD	220 (60.6)	485 (66.7)	705 (64.7)	0.006
Newly diagnosed PD	201 (55.4)	399 (54.9)	600 (55.0)	0.930
Prior SRB, median (min; max)	1 (1; 10)	1 (1; 86)	1 (1; 86)	0.007
Missing	14	40	54	
Intention				
Suicidal	300 (82.6)	532 (73.2)	832 (76.3)	< 0.001
Parasuicidal	63 (17.4)	195 (26.8)	258 (23.7)	
Self-injury	35 (9.6)	62 (8.5)	97 (8.9)	0.573
Family disposition	10 (2.8)	19 (2.6)	29 (2.7)	1.000
Suicidal ideation	82 (22.6)	155 (21.3)	237 (21.7)	0.689
Suicide announcement				
Written	51 (14.0)	96 (13.2)	147 (13.5)	0.776
Oral	74 (20.4)	161 (22.1)	235 (21.6)	
Farewell letter	55 (15.2)	101 (13.9)	156 (14.3)	0.640
Distancing, first psychiatric exploration	181 (53.4)	401 (57.5)	582 (56.2)	0.233
Missing	24	30	54	
Distancing, second psychiatric exploration	139 (64.7)	288 (69.2)	427 (67.7)	0.282
Missing	148	311	459	
Follow-up therapy				
Outpatient psychiatric care	9 (2.5)	22 (3.0)	31 (2.8)	0.001
Inpatient psychiatric care	231 (63.6)	378 (52.0)	609 (55.9)	
Discharge against medical advice	76 (20.9)	210 (28.9)	286 (26.2)	
Discharged home	33 (9.1)	103 (14.2)	136 (12.5)	
Other therapy	7 (1.9)	9 (1.2)	16 (1.5)	
Deceased	7 (1.9)	5 (0.7)	12 (1.1)	

Data are *n* (%), unless otherwise indicated. Percentages may not total 100% due to rounding.

*PD* psychiatric disorder, *SRB* suicide-related behavior

**Table 2:** Patient characteristics according to age group

	< 18 years *n* = 58 (5.3%)	18–44 years *n* = 603 (55.3%)	45–64 years *n* = 318 (29.2%)	> 64 years *n* = 111 (10.2%)	Total *n* = 1090 (100.0%)	*p*-value
Known PD	35 (60.3)	385 (63.8)	224 (70.4)	61 (55.0)	705 (64.7)	0.020
Newly diagnosed PD	33 (56.9)	328 (54.4)	176 (55.3)	63 (56.8)	600 (55.0)	0.955
Prior SRB, median (min; max)	1 (1; 5)	1 (1; 86)	1 (0; 10)	1 (1; 7)	1036 (1 (1; 86)	0.147
Missing	3	36	12	3	54	
Intention						
Suicidal	43 (74.1)	427 (70.8)	262 (82.4)	100 (90.1)	832 (76.3)	< 0.001
Parasuicidal	15 (25.9)	176 (29.3)	56 (17.6)	11 (9.9)	258 (23.7)	
Self-injury	13 (22.4)	54 (9.0)	22 (6.9)	8 (7.2)	97 (8.9)	0.006
Family disposition	2 (3.4)	13 (2.2)	11 (3.5)	3 (2.7)	29 (2.7)	0.560
Suicidal ideation	16 (27.6)	125 (20.7)	71 (22.3)	25 (22.5)	237 (21.7)	0.652
Suicide announcement						
Written	10 (17.2)	78 (12.9)	41 (12.9)	18 (16.2)	147 (13.5)	0.047
Oral	18 (31.0)	142 (23.5)	61 (19.2)	14 (12.6)	235 (21.6)	
Farewell letter	10 (17.2)	72 (11.9)	43 (13.5)	31 (27.9)	156 (14.3)	< 0.001
Distancing, first psychiatric exploration	23 (42.6)	349 (60.1)	158 (52.3)	52 (52.5)	582 (56.2)	0.019
Missing	4	22	16	12	54	
Distancing, second psychiatric exploration	11 (42.3)	246 (70.1)	123 (68.0)	47 (64.4)	427 (67.7)	0.030
Missing	32	252	137	38	459	
Follow-up therapy						
Outpatient psychiatric care	1 (1.7)	23 (3.8)	4 (1.3)	3 (2.7)	31 (2.8)	< 0.001
Inpatient psychiatric care	43 (74.1)	303 (50.2)	191 (60.1)	72 (64.9)	609 (55.9)	
Discharge against medical advice	6 (10.3)	191 (31.7)	78 (24.5)	11 (9.9)	286 (26.2)	
Discharged home	7 (12.1)	84 (13.9)	34 (10.7)	14 (12.6)	136 (12.5)	
Other therapy	0 (0)	4 (0.7)	6 (1.9)	6 (5.4)	16 (1.5)	
Deceased	1 (1.7)	1 (0.2)	5 (1.6)	5 (4.5)	12 (1.1)	

Data are *n* (%), unless otherwise indicated. Percentages may not total 100% due to rounding

*PD* psychiatric disorder, *SRB* suicide-related behavior

Additionally, we observed differences between the age groups with respect to the number of patients with known PD, intention, self-harm, suicide announcement, farewell letter, distancing from suicide at first and at second psychiatric exploration and follow-up therapy (Table 2).

The frequency of suicidal (versus parasuicidal) intent was greater among the male and in patients aged over 64 years. Furthermore, patients in that age group more frequently left a suicide note. Patients in age group > 64 and those younger than 18 years were more often transferred to further inpatient psychiatric treatment than those in other age groups. Follow-up therapy involving inpatient psychiatric treatment was also more often received by males than females. In the patients aged < 18 years, suicide attempt was significantly more often accompanied by self-injurious behavior than in all other age groups. These patients least frequently distanced themselves from suicidality in the first psychiatric exploration. Interestingly, the proportion of patients that distanced themselves from SRB did not increase among the youngest patients at the second survey which contrasted with all other age groups.

Thirty-seven patients had at least one more hospitalization due to self-poisoning (50 cases of multiple admissions in total, including 24 patients with one more hospitalization and 13 patients who were admitted two more times). Seven out of these 37 patients were male (18.9%); 30 patients had a pre-existing PD (81.1%). Number of substances, clinical outcome, distance from suicidality, and follow-up therapy were comparable between the first and the last admission in these 37 patients (Additional file 1).

### Circumstances of suicide-related behavior

Most patients were hospitalized due to SRB in spring and winter (see Additional file 2). In one-third of patients, SRB occurred between noon and 21:00, time of SRB was unknown for more than another third of patients. SRB occurred most often on Tuesday and least frequently on Wednesday. The predominant site of SRB was predominantly the patient´s home (see Additional file 2).

### Relationship between known psychiatric disorder and patient characteristics

The correlation between pre-existing PD and patient characteristics is shown in Additional file 3. Compared with patients without a known PD, those with pre-existing PD had already attempted suicide at least twice (40.7% vs 15.3%; *p* < 0.001) and ingested more substances on average (1.95 vs 1.79; *p* = 0.001). Patients with pre-existing PD predominantly ingested their own long-term psychiatric medications (70.2% vs 38.9% in those without known PD); patients without pre-existing PD also frequently used over-the-counter medications or home pharmacy medications (25.9%, *p* < 0.001). This tendency is also reflected in substances used by the respective group: while patients with pre-existing PD most often ingested antidepressants (37.2%), benzodiazepines (27.2%) and antipsychotics (25.4%), patients without known PD preferred OTC agents, such as non-opioid analgesics (34.8%) and antihistamines (11.7%), and benzodiazepines (19.5%). 38.7% of patients with PD did not report a specific trigger for the current suicide attempt; no trigger could be identified in only 18.1% of those without PD (*p* < 0.001). Among patients with identifiable trigger, partnership conflicts dominated in patients without PD (41.6%), whereas it was reported in a lower proportion of patients with PD (25.6%). Patients with PD were significantly less likely to distance themselves from the current suicide attempt in both the first and second psychiatric explorations (50.8% vs 65.9% and 62.9% vs 76.2% in those without known PD; *p* < 0.001, both) and, accordingly, were more likely to be referred to further inpatient psychiatric treatment (62.8% vs 43.1%; *p* < 0.001). In contrast, patients without pre-existing PD were more often discharged home regularly (18.4%) or against medical advice (30.6%) than those with pre-existing PD (9.2% vs 23.8%).

### Relationship between different psychiatric disorders and patient characteristics

Patients were categorized into the following PD categories: addiction (*n* = 31; 2.8%), schizophrenia (*n* = 52; 4.8%), mood disorder (*n* = 324; 29.7%), stress disorder (*n* = 185; 17.0%), personality disorder (*n* = 67; 6.1%), other PD (*n* = 9; 0.8%), and combined PD (*n* = 357; 32.8%). In combined PD, mood disorders were most frequent (32.6%), followed by personality disorders (18.4%), stress disorders (18.2%) and addictive disorders (16.4%, see Additional file 4). Sixty-five patients (6.0%) had no PD. Associations between different psychiatric disorders and various parameters are listed in Table 3.

**Table 3:** Relationship between psychiatric disorders and different variables

	Addiction *n* = 31 (2.8%)	Schizophrenia *n* = 52 (4.8%)	Mood disorder *n* = 324 (29.7%)	Stress disorder *n* = 185 (17.0%)	Personality disorder *n* = 67 (6.1%)	Other PD *n* = 9 (0.8%)	Combined PD *n* = 357 (32.8%)	No PD *n* = 65 (6.0%)	Total *n* = 1090 (100.0%)	*p*-value
Sex										
Male	19 (61.3)	23 (44.2)	103 (31.8)	67 (36.2)	15 (22.4)	1 (11.1)	111 (31.1)	24 (38.7)	363 (33.3)	0.004
Female	12 (38.7)	29 (55.8)	221 (68.2)	118 (63.8)	52 (77.6)	8 (88.9)	246 (68.9)	41 (63.1)	727 (66.7)	
Age group										
< 18	1 (3.2)	0 (0)	10 (3.1)	16 (8.6)	7 (10.4)	4 (44.4)	18 (5.0)	2 (3.1)	58 (5.3)	< 0.001
18–44	18 (58.1)	31 (59.6)	141 (43.5)	116 (62.7)	56 (83.6)	1 (11.1)	209 (58.5)	31 (47.7)	603 (55.3)	
45–64	10 (32.3)	18 (34.6)	123 (38.0)	36 (19.5)	4 (6.0)	1 (11.1)	107 (30.0)	19 (29.2)	318 (29.2)	
> 64	2 (6.5)	3 (5.8)	50 (15.4)	17 (9.2)	0 (0)	3 (33.3)	23 (6.4)	13 (20.0)	111 (10.2)	
Pre-existing psychiatric medication	5 (16.1)	42 (80.8)	170 (52.5)	10 (5.4)	32 (47.8)	4 (44.4)	245 (68.6)	3 (4.6)	511 (46.9)	< 0.001
Pre-existing psychiatric treatment	10 (12.3)	38 (73.1)	161 (49.7)	19 (10.3)	44 (65.7)	2 (22.2)	258 (72.3)	10 (15.4)	542 (49.7)	< 0.001
Prior SRB										
First SRB	30 (96.8)	37 (71.2)	223 (68.8)	156 (84.3)	37 (55.2)	8 (88.9)	194 (54.3)	56 (86.2)	741 (68.0)	< 0.001
At least second SRB	1 (3.2)	15 (28.8)	101 (31.2)	29 (15.7)	30 (44.8)	1 (11.1)	163 (45.7)	9 (13.8)	349 (32.0)	
Family disposition	0 (0)	1 (1.9)	14 (4.3)	2 (1.1)	0 (0)	1 (11.1)	10 (2.8)	1 (1.5)	29 (2.7)	0.171
Intention										
Suicidal	18 (58.1)	47 (90.4)	285 (88.0)	111 (60.0)	51 (76.1)	7 (77.8)	272 (76.2)	41 (63.1)	832 (76.3)	< 0.001
Parasuicidal	13 (41.9)	5 (9.6)	39 (12.0)	74 (40.0)	16 (23.9)	2 (22.2)	85 (23.8)	24 (36.9)	258 (23.7)	
Suicidal ideation	4 (12.9)	16 (30.8)	86 (26.5)	22 (11.9)	16 (23.9)	3 (33.3)	84 (23.5)	6 (9.2)	237 (21.7)	< 0.001
Suicide announcement										
Written	2 (6.5)	3 (5.8)	57 (17.6)	30 (16.2)	9 (13.4)	2 (22.2)	37 (10.4)	7 (10.8)	147 (13.5)	< 0.001
Oral	4 (12.9)	14 (26.9)	51 (15.7)	41 (22.2)	24 (35.8)	0 (0)	91 (25.5)	10 (15.4)	235 (21.6)	
Any announcement	6 (19.4)	17 (32.7)	108 (33.3)	71 (38.4)	33 (49.3)	2 (22.2)	128 (35.9)	17 (26.2)	382 (35.0)	
Farewell letter	1 (3.2)	5 (9.6)	59 (18.2)	24 (13.0)	6 (9.0)	2 (22.2)	48 (13.4)	11 (16.9)	156 (14.3)	0.129
Self-injury	3 (9.7)	3 (5.8)	24 (7.4)	9 (4.9)	11 (16.4)	0 (0)	40 (11.2)	7 (10.8)	97 (8.9)	0.066
Source of medication										
Next of kin/friend	1 (3.8)	1 (2.1)	14 (4.5)	15 (8.8)	5 (7.9)	1 (11.1)	5 (1.5)	2 (3.5)	44 (4.3)	< 0.001
Long-term/on demand	12 (46.2)	32 (68.1)	199 (64.2)	62 (36.5)	36 (57.1)	6 (66.7)	236 (69.0)	26 (45.6)	609 (59.5)	
Illegally obtained	4 (15.4)	0 (0)	0 (0)	2 (1.2)	0 (0)	0 (0)	2 (0.6)	0 (0)	8 (0.8)	
No medication	4 (15.4)	6 (12.8)	21 (6.8)	12 (7.1)	6 (9.5)	0 (0)	35 (10.2)	7 (12.3)	91 (8.9)	
Several sources	3 (11.5)	2 (4.3)	39 (12.6)	26 (15.3)	8 (12.7)	1 (11.1)	36 (10.5)	10 (17.5)	125 (12.2)	
OTC/pharmacy	1 (3.8)	6 (12.8)	33 (10.6)	53 (31.2)	8 (12.7)	1 (11.1)	24 (7.0)	12 (21.1)	138 (13.5)	
Other sources	1 (3.8)	0 (0)	4 (1.3)	0 (0)	0 (0)	0 (0)	4 (1.2)	0 (0)	9 (0.9)	
Missing	5	5	14	15	4	0	15	8	66	
Trigger										
Work	1 (3.3)	2 (3.9)	23 (7.2)	17 (9.2)	5 (7.6)	0 (0)	20 (5.7)	3 (4.7)	71 (6.6)	< 0.001
Family	3 (10.0)	1 (2.0)	32 (10.0)	20 (10.9)	4 (6.1)	3 (33.3)	29 (8.2)	9 (14.1)	101 (9.4)	
Financial problems	1 (3.3)	2 (3.9)	12 (3.8)	4 (2.2)	2 (3.0)	0 (0)	9 (2.5)	0 (0)	30 (2.8)	
Law/justice	1 (3.3)	1 (2.0)	7 (2.2)	6 (3.3)	0 (0)	0 (0)	7 (2.0)	1 (1.6)	23 (2.1)	
Health	2 (6.7)	3 (5.9)	30 (9.4)	10 (5.4)	1 (1.5)	0 (0)	33 (9.3)	4 (6.3)	83 (7.7)	
Partner	7 (23.3)	3 (5.9)	93 (29.1)	91 (49.5)	20 (30.3)	0 (0)	98 (27.8)	25 (39.1)	337 (31.3)	
Social environment	1 (3.3)	4 (7.8)	17 (5.3)	9 (4.9)	2 (3.0)	2 (22.2)	21 (5.9)	2 (3.1)	58 (5.4)	
Loss of attachment figure/pet animal	0 (0)	1 (2.0)	15 (4.7)	4 (2.2)	1 (1.5)	1 (11.1)	12 (3.4)	2 (3.1)	36 (3.3)	
No trigger	14 (46.7)	34 (66.7)	91 (28.4)	23 (12.5)	31 (47.0)	3 (33.3)	124 (35.1)	18 (28.1)	338 (31.4)	
Missing	1	1	4	1	1	0	4	1	13	
Severity										
None	3 (9.7)	7 (13.5)	43 (13.3)	45 (24.3)	17 (25.4)	1 (11.1)	62 (17.4)	15 (23.1)	193 (17.7)	< 0.001
Minor	22 (71.0)	22 (42.3)	148 (45.7)	101 (54.6)	33 (49.3)	5 (55.6)	199 (55.7)	28 (43.1)	558 (51.2)	
Moderate	3 (9.7)	19 (36.5)	104 (32.1)	32 (17.3)	14 (20.9)	2 (22.2)	75 (21.0)	15 (23.1)	264 (24.2)	
Severe	1 (3.2)	4 (7.7)	24 (7.4)	7 (3.8)	2 (3.0)	0 (0)	21 (5.9)	2 (3.1)	61 (5.6)	
Fatal	2 (6.5)	0 (0)	5 (1.5)	0 (0)	1 (1.5)	1 (11.1)	0 (0)	5 (7.7)	14 (1.3)	
Number of substances, mean; median (min; max)	1.68; 1.00 (1.00; 6.00)	1.60; 1.00 (1.00; 6.00)	1.92; 1.00 (1.00; 7.00)	1.77; 1.00 (1.00; 8.00)	1.93; 1.00 (1.00; 10.00)	3.00; 2.00 (1.00; 13.00)	1.99; 2.00 (1.00; 10.00)	1.77; 1.00 (1.00; 7.00)	1.90; 1.00 (1.00; 13.00)	0.040
Missing	0	0	1	1	0	0	1	1	4	
Substances										
Antibiotic	0 (0)	0 (0)	8 (2.5)	11 (5.9)	0 (0)	0 (0)	1 (0.3)	2 (3.1)	22 (2.0)	0.002
Anticoagulant	0 (0)	0 (0)	4 (1.2)	2 (1.1)	0 (0)	0 (0)	3 (0.8)	0 (0)	9 (0.8)	0.989
Anticonvulsant	5 (16.1)	8 (15.4)	22 (6.8)	14 (7.6)	9 (13.4)	0 (0)	32 (9.0)	4 (6.2)	94 (8.6)	0.191
Antidepressant	2 (6.5)	6 (11.5)	116 (35.8)	19 (10.3)	17 (25.4)	2 (22.2)	136 (38.1)	10 (15.4)	308 (28.3)	< 0.001
Antidiabetic	0 (0)	0 (0)	6 (1.9)	4 (2.2)	0 (0)	0 (0)	2 (0.6)	1 (1.5)	13 (1.2)	0.548
Antihistamine	0 (0)	5 (9.6)	21 (6.5)	20 (10.8)	5 (7.5)	0 (0)	19 (5.3)	6 (9.2)	76 (7.0)	0.206
Anti-Parkinson medication	0 (0)	1 (1.9)	2 (0.6)	2 (1.1)	1 (1.5)	1 (11.1)	2 (0.6)	0 (0)	9 (0.8)	0.172
Antipsychotics	2 (6.5)	19 (36.5)	50 (15.4)	3 (1.6)	17 (25.4)	0 (0)	97 (27.2)	3 (4.6)	191 (17.5)	< 0.001
Benzodiazepine	14 (45.2)	11 (21.2)	88 (27.2)	34 (18.4)	14 (20.9)	0 (0)	94 (26.3)	12 (18.5)	267 (24.5)	0.018
Car exhaust/carbon monoxide	1 (3.2)	0 (0)	9 (2.8)	2 (1.1)	1 (1.5)	0 (0)	4 (1.1)	3 (4.6)	20 (1.8)	0.310
Cardiac medication	0 (0)	4 (7.7)	29 (9.0)	15 (8.1)	1 (1.5)	1 (11.1)	14 (3.9)	3 (4.6)	67 (6.1)	0.034
Chemical	0 (0)	0 (0)	2 (0.6)	2 (1.1)	0 (0)	0 (0)	6 (1.7)	1 (1.5)	11 (1.0)	0.800
Cleaning agents	1 (3.2)	3 (5.8)	2 (0.6)	4 (2.2)	1 (1.5)	0 (0)	6 (1.7)	2 (3.1)	19 (1.7)	0.137
Cytostatics	0 (0)	0 (0)	1 (0.3)	0 (0)	0 (0)	0 (0)	2 (0.6)	0 (0)	3 (0.3)	0.901
Endocrinological medication	0 (0)	0 (0)	5 (1.5)	3 (1.6)	2 (3.0)	0 (0)	5 (1.4)	1 (1.5)	16 (1.5)	0.929
Fungicide	0 (0)	0 (0)	0 (0)	1 (0.5)	0 (0)	0 (0)	1 (0.3)	1 (1.5)	3 (0.3)	0.384
Herbal medicine	1 (3.2)	2 (3.8)	7 (2.2)	2 (1.1)	1 (1.5)	0 (0)	2 (0.6)	3 (4.6)	18 (1.7)	0.100
(Illegal) drugs	4 (12.9)	1 (1.9)	3 (0.9)	4 (2.2)	5 (7.5)	0 (0)	24 (6.7)	1 (1.5)	42 (3.9)	< 0.001
Insecticide	0 (0)	0 (0)	2 (0.6)	2 (1.1)	0 (0)	0 (0)	2 (0.6)	0 (0)	6 (0.6)	0.94
Mushrooms	0 (0)	0 (0)	0 (0)	0 (0)	0 (0)	0 (0)	1 (0.3)	0 (0)	0 (0)	1.000
Non-opioid analgesics	4 (12.9)	3 (5.8)	75 (23.1)	79 (42.7)	18 (26.9)	5 (55.6)	64 (17.9)	16 (24.6)	264 (24.2)	< 0.001
Opioids	5 (16.1)	1 (1.9)	20 (6.2)	14 (7.6)	3 (4.5)	1 (11.1)	26 (7.3)	4 (6.2)	74 (6.8)	0.351
Other drugs	2 (6.5)	1 (1.9)	19 (5.9)	15 (8.1)	5 (7.5)	2 (22.2)	29 (8.1)	8 (12.3)	81 (7.4)	0.217
Other sedatives	2 (6.5)	0 (0)	1 (0.3)	3 (1.6)	0 (0)	0 (0)	1 (0.3)	0 (0)	7 (0.6)	0.046
Other substances	0 (0)	0 (0)	1 (0.3)	0 (0)	1 (1.5)	0 (0)	1 (0.3)	1 (2)	4 (0.4)	0.332
Plant	0 (0)	1 (1.9)	2 (0.6)	0 (0)	1 (1.5)	0 (0)	3 (0.8)	0 (0)	7 (0.6)	0.512
Rodenticides	0 (0)	1 (1.9)	2 (0.6)	1 (0.5)	0 (0)	0 (0)	0 (0)	1 (1.5)	5 (0.5)	0.201
Z-drugs	5 (16.1)	7 (13.5)	54 (16.7)	21 (11.4)	8 (11.9)	2 (22.2)	41 (11.5)	9 (13.8)	147 (13.5)	0.512
Distancing, first psychiatric exploration	19 (70.4)	15 (31.2)	149 (47.9)	132 (74.2)	37 (56.9)	5 (55.6)	182 (52.9)	43 (79.6)	582 (56.2)	< 0.001
Missing	4	4	13	7	2	0	13	11	54	
Distancing, second psychiatric exploration	14 (82.4)	14 (43.8)	133 (63.3)	89 (85.6)	29 (72.5)	3 (50.0)	128 (64.0)	17 (77.3)	427 (67.7)	< 0.001
Missing	14	20	114	81	27	3	157	43	459	
Follow-up therapy										
Outpatient psychiatric care	3 (9.7)	0 (0)	10 (3.1)	9 (4.9)	3 (4.5)	0 (0)	6 (1.7)	0 (0)	31 (2.8)	< 0.001
Inpatient psychiatric care	5 (16.1)	48 (92.3)	223 (68.8)	54 (29.2)	40 (59.7)	5 (55.6)	216 (60.5)	18 (27.7)	609 (55.9)	
Discharge against medical advice	16 (51.6)	2 (3.8)	50 (15.4)	75 (40.5)	17 (25.4)	1 (11.1)	101 (28.3)	24 (36.9)	286 (26.2)	
Discharged home	3 (9.7)	2 (3.8)	33 (10.2)	47 (25.4)	6 (9.0)	2 (22.2)	31 (8.7)	12 (18.5)	136 (12.5)	
Other therapy	3 (9.7)	0 (0)	4 (1.2)	0 (0)	0 (0)	0 (0)	3 (0.8)	6 (9.2)	16 (1.5)	
Deceased	1 (3.2)	0 (0)	4 (1.2)	0 (0)	1 (1.5)	1 (11.1)	0 (0)	5 (7.7)	12 (1.1)	

Data are *n* (%), unless otherwise indicated. Percentages may not total 100% due to rounding

*OTC* over-the-counter medication, *PD* psychiatric disorder, *SRB* suicide-related behavior

Percentage of patients with each PD differed in terms of sex, age group, prior psychiatric care and medication, number of prior SRB, intention, suicidal ideation and announcement, source and type of medication, trigger, severity, distancing from suicide ideation and type of follow-up therapy. Apart from addictive disorders, where males predominated at 61.3%, all other PDs were dominated by the female gender. Except for “other psychiatric disorders”, the 18–44 age group represented the largest age group for all PDs, especially personality disorders at 83.6%. Although more than half of patients were hospitalized due to their first SRB, a particularly high proportion of suicide attempt recurrences was found among patients with combined PD and personality disorders. The schizophrenic and mood disorders had a particularly high proportion of suicidal intent (90.4% and 88%, respectively), compared to stress disorders and addiction (60.0% and 58.1%, respectively), and those with no PD (63.1%). Most patients had no suicidal ideation; however, it was documented in about a third of patients with schizophrenia and those with other PD. Similarly, most patients did not announce the suicide, however, almost half of those with personality disorder announced the suicide attempt. By far, the most common source of medication was the patient’s own long-term medication for all PDs except for the stress disorder group, for which after the patient's own medication OTC medication or the medicine cabinet served as the source for nearly one-third. This pattern is also reflected in the most frequently used substance groups: antidepressants were most often used in patients with mood disorder and combined PD, while antipsychotics were preferentially ingested in patients with schizophrenia followed by patients with personality disorder and those with combined PD. Patients with addiction more often than others used benzodiazepines, (illegal) drugs and other sedatives, while patients with stress disorder and those with other PD most often took non-opioid analgesics. It is noticeable that a high percentage of patients with schizophrenia, personality disorders, addiction, and combined PD did not have a detectable trigger. Conversely, partnership conflicts predominated by far in patients with stress disorders. The severity of poisoning according to PSS was most frequently minor except for schizophrenia and mood disorder where a high proportion of moderate and severe poisonings was observed. Apart from those with schizophrenia and affective disorders, most patients in other PD groups already distanced themselves from suicidality at the first interview. The proportion of those distanced from suicidality increased at the second exploration in all PD groups except in the group with other PD and in those without PD. With regard to follow-up therapy, the vast majority of patients with schizophrenia, mood disorder, personality disorder and other or combined PD were admitted to inpatient psychiatric therapy. In contrast, the remaining patients with addiction, stress disorder or no PD were most often discharged against medical advice.

## Discussion

In this retrospective analysis of a relatively large cohort of patients, we aimed to characterize factors associated with suicidal self-poisoning. We hoped that results of our study would help to determine risk constellations which could be utilized to identify subgroups at a higher risk of suicide to which more precise prevention strategies can be tailored.

The main findings of our study are: (i) two-thirds of the patients were women and more than half of the patients (55.3%) were between 18 and 44 years old; (ii) in almost one-third of the patients, no detectable trigger could be found; in particular, patients with PD did not report a specific trigger for the suicide attempt compared to patients without PD (38.7% vs 18.1%); (iii) 64.7% of patients had a pre-existing PD and these patients ingested a higher number of substances overall, which most often included their long-term psychiatric medication; (iv) a pre-existing PD had no influence on the severity of the intoxication although moderate to severe courses of intoxication were predominantly documented in schizophrenic and mood disorders, while asymptomatic and mild courses dominated in patients with stress and personality disorders; (v) patients with a history of PD significantly more often displayed prior SRB, with recurrence particularly common in patients with combined PD (45.7%) and personality disorder (44.8%); (vi) patients with pre-existing PD were significantly more likely to receive further inpatient psychiatric treatment (particularly in the schizophrenic disorders group (92.3%)).

In line with previous analyses, most of our SRB cases were documented in females [4, 23–26]. Importantly, a trigger could not be identified in approximately one-third of all cases, particularly among patients with a known PD, which could contribute to difficulties in identifying of individuals at risk of SRB. In the remaining patients, SRB was more frequently triggered by issues related to conflicts with partner, or family dispute and health problems. Patients with stress disorder were the only group with a dominant identifiable trigger, namely partnership conflicts. Patients in this group received neither prior psychiatric care nor medication and most of them attempted suicide for the first time. Of note, patients with stress disorder distanced themselves from SRB more often than remaining patients.

Two-thirds of cases had a known PD which supports previous observations of a high rate of self-poisoning attempted suicide in patients with psychiatric treatment [27]. These patients more often had at least one prior SRB, displayed suicidal rather than parasuicidal behavior and had a suicidal ideation more often than those with unknown PD. Beautrais et al. found an increased rate of psychiatric illness at the time of SRB in young people with severe outcome [28].

A vast majority of patients used their medication rather than other type of substance which is in line with prior findings from a Swiss study investigating suicide attempts [28]. Patients with previous PD used significantly more substances than the group with no PD. Consistent with the substance source findings in this and other studies [29, 30], long-term and on demand medications (most common among these antidepressants, benzodiazepines, antipsychotics) were far more frequently chosen by patients with a known PD. On the contrary, patients without pre-existing PD more often self-intoxicated with the OTC medication, potentially “leftovers" from the medicine cabinet. Among these medications, especially antidepressants are a known risk factor for SRB which is most pronounced in youth and during the first weeks of therapy [27, 31]. Overall, our findings confirm the results from a prior meta-analyses demonstrating that female sex, age 15–40 years, psychiatric disorder and psychiatric treatment using antidepressants are among the main risk factors for suicide by poisoning [32].

While Frei et al. showed that suicide announcement was frequent among patients with PD [33], we did not observe a difference in rate of suicide announcement between patients with and without known PD. We also found that 14.3% of patients left a suicide note which was about half as often as in a Japanese study of completed suicides [34]. That study also demonstrated that patients who committed suicide due to psychiatric illness tended to write a suicide note less frequently [34] in line with other investigators who showed that suicide victims without a psychiatric history leave a suicide note more often [35].

Patients in our study most often suffered from a mood disorder, followed by stress disorder, personality disorder, schizophrenia, and addiction. In a previous study involving predominantly deliberate self-poisoning cases in Turkey, 28.5% patients had depressive disorder [36]. Depression, stress disorder and personality disorder were the most frequent three diagnoses among the patients with self-harm in Switzerland [28]. Mood disorder has been previously identified as a risk factor for deliberate self-poisoning [37]. Patients with bipolar affective disorder are at particularly high risk of suicide which is estimated to be 17 to 20 times higher than in the general population [38]. Furthermore, psychiatric autopsies reported in several studies demonstrated that suicide completers are more likely to have more than one psychiatric diagnosis [39, 40]. In contrast, in our study, there was not a single fatality and only a small proportion of severe poisonings (5.9%) in the combined PD group. We found patients with combined PD and those with mood disorder in our study most often used long-term/on demand medication and had minor to moderate PSS. Approximately a quarter of patients with combined PD and those with mood disorder had suicidal ideation before the current suicide attempt. The differences between these two groups included a higher percentage of patients with two or more SRB and prior psychiatric care and medication in those with combined PD, while those with mood disorder more frequently displayed suicidal intent. These findings are similar to a previous study in male prisoners showing that major depression followed by psychosis, anxiety and addictive disorders are associated with a severe suicide attempt [41].

Patients with schizophrenia had a higher severity of poisoning, they received prior psychiatric care and medication, and preferably used their long-term/on demand medicines. An increased risk of suicide in schizophrenia or schizoaffective disorder is well known and has been estimated to have a lifetime prevalence of 5.6% [42]. Additionally, patients with schizophrenia did not distance themselves from suicide ideation after first SRB and had the highest rate of admission to inpatient psychiatric care. It was previously shown that patients with schizophrenia or other psychotic disorder were most likely to be discharged to a psychiatric hospital after deliberate self-poisoning [43].

Addiction was the only group including more males than females, thus corroborating the previous results in patients with self-poisoning SRB [37, 44]. Prior psychiatric care or medication was infrequent and almost all patients in this group attempted the suicide for the first time. Most patients had no detectable trigger and did not announce the suicide. Interestingly, patients with addiction distanced themselves more often after second than after first psychiatric exploration.

More than half of our patients were admitted to the inpatient psychiatric care. Inpatient care group included relatively more males than in other types of follow-up, and most patients suffered from mood disorder or combined disorder. The PSS was high and almost every patient displayed suicidal behavior. Compared to our study, other authors reported lower rates of institutionalization after deliberate self-poisoning. For example, Bjornaas et al. found that only 19.0% of patients were institutionalized after SRB [45]. Furthermore, Carter et al. reported that 29.2% of patients were referred to psychiatric hospital [43], whereas 65.2% were discharged home or received outpatient care after SRB involving self-poisoning. Moreover, these studies found that a much fewer patients were discharged against medical advice than in our study (2.0%-3.0% vs 26.2%, [43, 45]). However, these discrepancies could be explained by the striking differences in health care provision in the respective countries. In line with our study, these authors reported that patients referred to inpatient care were more often male and suffered from mood disorder. The most frequent diagnoses among fatal cases were other and no psychiatric disorders. The latter supports the results of two Swiss studies, which showed that the majority of suicidal patients had not been previously diagnosed [26, 33].

### Limitations

The limitations of our work include the retrospective nature of the study, the monocentric setting, and a suspected sampling bias. Since the survey was limited to the inpatient-somatic stay of the patients, a long-term follow-up of the patients is lacking. Furthermore, a certain degree of imprecision must be assumed as the information on the ingested substances (if no toxicological analysis was performed), pre-existing PDs or pre-existing psychiatric medication, and details on circumstances of the suicide were often based on incomplete information provided by the patients or on information obtained from external anamneses. Another limitation is that we did not use standardized scales or questionnaires to distinct between suicidal and parasuicidal behavior or to evaluate the degree of suicidal intent [46]. Moreover, in a very few patients without psychiatric exploration, the discrimination between suicidal and parasuicidal intent was made by the admitting physicians who are less trained in recognizing the motivation for SRB. For example, admitting physicians were more likely to associate ingestion a very small dose of a substance or discharge home with parasuicidal rather than suicidal act. Therefore, there is a risk that determination of intent could have been imprecise in patients without psychiatric interview. Finally, since the data collection was not hypothesis-driven, but it was rather aimed at capturing the interrelationships of a variety of parameters, random findings may have arisen due to multiple testing. Therefore, statistically significant results should be interpreted as trends until they will be validated in separate confirmatory studies.

Beside important limitations, our study has also strengths. In addition to the relatively large number of patients, the particular clinical setting that allows the combination of internal medicine–toxicology-guided treatment in close cooperation with a psychiatrist should be emphasized. Most of the studies either focus on clinical–toxicological details or focus on psychiatric aspects without involving the other discipline in each case. Our interdisciplinary approach allowed the inclusion of a very broad spectrum of patients ranging from clinically asymptomatic to lethal poisonings. All patients received toxicological or intensive medical treatment tailored to the respective poisoning pattern. Subsequently, patients with only mild psychiatric disorders or in crisis situations were discharged to home or to further outpatient psychiatric care while those with severe psychiatric disorders were transferred directly to further inpatient psychiatric therapy for early psychiatric co-care.

### Conclusion

This study identified female gender, age between 18 and 44 years, PD (particularly schizophrenia or mood disorder), history of attempted suicide and distancing from suicide as factors associated with increased risk for suicidal self-poisoning. Results of this study could be used to further refine the suicide risk assessment by psychiatrists and other treating physicians, who should be particularly sensitized to patients with this risk constellation. These patients often suffer from a severe course of intoxication, possibly due to the choice of potentially more harmful substances from their own psychiatric long-term medication. Lack of a detectable trigger in patients with a known PD could hinder identification of individuals at risk of SRB, and these patients should be evaluated more frequently. From the perspective of practical implications—detecting a profile at risk for a specific suicide method, namely self-poisoning, could help to develop more tailored preventive measures towards vulnerable subgroups.

## Supplementary Information

**Additional file 1.** Characteristics of patients with multiple admissions.

**Additional file 2.** Circumstances of the suicide-related behavior, including (a) season of the year, (b) time of day, (c) day of the week and (d) site.

**Additional file 3.** Relationship between known psychiatric disorder and different variables.

**Additional file 4.** Distribution of different psychiatric disorders among the patients classified into the combined psychiatric disorder group.

## Data Availability

Data used for this analysis are available upon reasonable request to the corresponding author.
