# Supplementary material for: Socio-demographic and psychiatric profile of patients hospitalized due to self-poisoning with suicidal intention
Source: Ann Gen Psychiatry. 2022 Jun 9;21:16. doi: 10.1186/s12991-022-00393-3 (PMC9185897; doi:10.1186/s12991-022-00393-3)
Supplement: Supplementary file 1 — Additional file 1. Characteristics of patients with multiple admissions. [file 12991_2022_393_MOESM1_ESM.docx]

Additional file 1: Characteristics of patients with multiple admissions.

|  | **First admission**  **n=37**  **(100%)** | **Last admission**  **n=37**  **(100%)** | **p-value** |
| --- | --- | --- | --- |
| **Number of substances** |  |  |  |
| 1 | 16 (43.2) | 20 (54.1) | 0.759 |
| 2 | 8 (21.6) | 7 (18.9) |  |
| ≥3 | 13 (35.1) | 10 (27.0) |  |
| **Severity** |  |  |  |
| None | 7 (18.9) | 5 (13.5) | 0.700 |
| Minor | 22 (59.5) | 25 (67.6) |  |
| Moderate | 5 (13.5) | 6 (16.2) |  |
| Severe | 3 (8.0) | 1 (2.7) |  |
| **Distancing from suicide** | 14 (37.8) | 16 (43.2) | 0.953 |
| Unknown | 16 | 14 |  |
| **Follow-up therapy** |  |  |  |
| Inpatient psychiatric care | 20 (54.1) | 19 (51.4) | 0.304 |
| Discharge against medical advice | 10 (27.0) | 14 (37.8) |  |
| Discharged home | 5 (13.5) | 2 (5.4) |  |
| Other therapy | 2 (5.4) | 1 (2.7) |  |
| Missing | 0 | 1 |  |

Data are n (%), unless otherwise indicated. Percentages may not total 100% due to rounding.
