# Supplementary material for: Socio-demographic and psychiatric profile of patients hospitalized due to self-poisoning with suicidal intention
Source: Ann Gen Psychiatry. 2022 Jun 9;21:16. doi: 10.1186/s12991-022-00393-3 (PMC9185897; doi:10.1186/s12991-022-00393-3)
Supplement: Supplementary file 2 — Additional file 2. Circumstances of the suicide-related behavior, including (a) season of the year, (b) time of day, (c) day of the week and (d) site. [file 12991_2022_393_MOESM2_ESM.docx]

Additional file 2: Circumstances of the suicide-related behavior, including (a) season of the year, (b) time of day, (c) day of the week and (d) site.

|   **c** |   **b** |
| --- | --- |
|  |   **d** |
