# Supplementary material for: Socio-demographic and psychiatric profile of patients hospitalized due to self-poisoning with suicidal intention
Source: Ann Gen Psychiatry. 2022 Jun 9;21:16. doi: 10.1186/s12991-022-00393-3 (PMC9185897; doi:10.1186/s12991-022-00393-3)
Supplement: Supplementary file 3 — Additional file 3. Relationship between known psychiatric disorder and different variables. [file 12991_2022_393_MOESM3_ESM.docx]

Additional file 3: Relationship between known psychiatric disorder and different variables.

|  | **Psychiatric disorder known (n=705; 64.7%)** | **Psychiatric disorder unknown (n=385; 35.3%)** | **Total**  **(n=1090; 100%)** | **p-value** |
| --- | --- | --- | --- | --- |
| **Sex** |  |  |  |  |
| Male | 220 (31.2) | 143 (37.1) | 363 (33.3) | 0.055 |
| Female | 485 (68.8) | 242 (62.9) | 727 (66.7) |  |
| **Age groups** |  |  |  |  |
| <18 | 35 (5.0) | 23 (6.0) | 58 (5.3) | 0.02 |
| 18-44 | 385 (54.6) | 218 (56.6) | 603 (55.3) |  |
| 45-64 | 224 (31.8) | 94 (24.4) | 318 (29.2) |  |
| >64 | 61 (8.7) | 50 (13.0) | 111 (10.2) |  |
| **Pre-existing psychiatric medication** | 482 (68.4) | 29 (7.5) | 511 (46.9) | <0.001 |
| **Pre-existing psychiatric treatment** | 507 (71.9) | 35 (9.1) | 542 (49.7) | <0.001 |
| **Prior SRB** |  |  |  |  |
| First SRB | 418 (59.3) | 326 (84.7) | 744 (68.3) | <0.001 |
| At least second SRB | 287 (40.7) | 59 (15.3) | 346 (31.7) |  |
| **Family disposition** | 23 (3.3) | 6 (1.6) | 29 (2.7) | 0.115 |
| **Intention** |  |  |  |  |
| Suicidal | 559 (79.3) | 273 (70.9) | 832 (76.3) | 0.002 |
| Parasuicidal | 146 (20.7) | 112 (29.1) | 258 (23.7) |  |
| **Suicidal thoughts** | 167 (23.7) | 70 (18.2) | 237 (21.7) | 0.038 |
| **Suicide announcement** |  |  |  |  |
| Written | 86 (12.2) | 61 (15.8) | 147 (13.5) | 0.108 |
| Oral | 162 (23.0) | 73 (19.0) | 235 (21.6) |  |
| Any announcement | 248 (35.2) | 134 (34.8) | 382 (35.0) |  |
| **Farewell letter** | 94 (13.3) | 62 (16.1) | 156 (14.3) | 0.239 |
| **Self-harm** | 68 (9.6) | 29 (7.5) | 97 (8.9) | 0.267 |
| **Source of medication** |  |  |  |  |
| Next of kin/friend | 13 (1.9) | 31 (8.8) | 44 (4.3) | <0.001 |
| Long-term/on demand | 472 (70.2) | 137 (38.9) | 609 (59.5) |  |
| Illegally obtained | 5 (0.7) | 3 (0.9) | 8 (0.8) |  |
| No medication | 55 (8.2) | 36 (10.2) | 91 (8.9) |  |
| Several sources | 74 (11.0) | 51 (14.5) | 125 (12.2) |  |
| OTC/pharmacy | 47 (7.0) | 91 (25.9) | 138 (13.5) |  |
| Other sources | 6 (0.9) | 3 (0.3) | 9 (0.9) |  |
| Missing | 33 | 33 | 66 |  |
| **Trigger** |  |  |  |  |
| Work | 43 (6.2) | 28 (7.3) | 71 (6.6) | <0.001 |
| Family | 53 (7.6) | 48 (12.6) | 101 (9.4) |  |
| Financial problems | 15 (2.2) | 15 (3.9) | 30 (2.8) |  |
| Law/Justice | 16 (2.3) | 7 (1.8) | 23 (2.1) |  |
| Health | 56 (8.1) | 27 (7.1) | 83 (7.7) |  |
| No trigger | 269 (38.7) | 69 (18.1) | 338 (31.4) |  |
| Partner | 178 (25.6) | 159 (41.6) | 337 (31.3) |  |
| Social environment | 41 (5.9) | 17 (4.5) | 58 (5.4) |  |
| Loss of attachment figure/pet animal | 24 (3.5) | 12 (3.1) | 36 (3.3) |  |
| Missing | 10 | 3 | 13 |  |
| **Severity** |  |  |  |  |
| None | 112 (15.9) | 81 (21.0) | 193 (17.7) | 0.12 |
| Minor | 376 (53.3) | 182 (47.3) | 558 (51.2) |  |
| Moderate | 166 (23.5) | 98 (25.5) | 264 (24.2) |  |
| Severe | 43 (6.1) | 18 (4.7) | 61 (5.6) |  |
| Fatal | 8 (1.1) | 6 (1.6) | 14 (1.3) |  |
| **Number of substances, mean; median (min; max)** | 1.95; 1.00 (0.00; 10.00) | 1.79; 1.00 (1.00; 13.00) | 1.90; 1.00  1.00; 13.00) | 0.001 |
| Missing | 3 | 1 | 4 |  |
| **Substances** |  |  |  |  |
| Antibiotic | 3 (0.4) | 19 (4.9) | 22 (2.0) | <0.001 |
| Anticoagulant | 6 (0.9) | 3 (0.8) | 9 (0.8) | 1 |
| Anticonvulsant | 63 (8.9) | 31 (8.1) | 94 (8.6) | 0.653 |
| Antidepressant | 262 (37.2) | 46 (11.9) | 308 (28.3) | <0.001 |
| Antidiabetic | 5 (0.7) | 8 (2.1) | 13 (1.2) | 0.075 |
| Antihistamine | 31 (4.4) | 45 (11.7) | 76 (7.0) | <0.001 |
| Anti-Parkinson medication | 6 (0.9) | 3 (0.8) | 9 (0.8) | 1 |
| Antipsychotics | 179 (25.4) | 12 (3.1) | 191 (17.5) | <0.001 |
| Benzodiazepine | 192 (27.2) | 75 (19.5) | 267 (24.5) | 0.005 |
| Car exhaust/Carbon monoxide | 9 (1.3) | 11 (2.9) | 20 (1.8) | 0.095 |
| Cardiac medication | 41 (5.8) | 26 (6.8) | 67 (6.1) | 0.598 |
| Chemical | 6 (0.9) | 5 (1.3) | 11 (1.0) | 0.532 |
| Cleaning agents | 10 (1.4) | 9 (2.3) | 19 (1.7) | 0.333 |
| Cytostatics | 2 (0.3) | 1 (0.3) | 3 (0.3) |  |
| Endocrinological medication | 9 (1.3) | 7 (1.8) | 16 (1.5) | 0.599 |
| Fungicide | 1 (0.1) | 2 (0.5) | 3 (0.3) | 0.286 |
| Herbal medicine | 10 (1.4) | 8 (2.1) | 18 (1.7) | 0.459 |
| (Illegal) drugs | 32 (4.5) | 10 (2.6) | 42 (3.9) | 0.138 |
| Insecticides | 3 (0.4) | 3 (0.8) | 6 (0.6) | 0.432 |
| Mushrooms | 1 (0.1) | 0 (0) | 1 (0.1) | 1 |
| Non-opioid analgesics | 130 (18.4) | 134 (34.8) | 264 (24.2) | <0.001 |
| Opioids | 43 (6.1) | 31 (8.1) | 74 (6.8) | 0.257 |
| Other drugs | 47 (6.7) | 34 (8.8) | 81 (7.4) | 0.227 |
| Other sedatives | 4 (0.6) | 3 (0.8) | 7 (0.6) | 0.703 |
| Other substances | 3 (0.4) | 1 (0.3) | 4 (0.4) | 1 |
| Plants | 7 (1.0) | 0 (0) | 7 (0.6) | 0.056 |
| Rodenticides | 2 (0.3) | 3 (0.8) | 5 (0.5) | 0.352 |
| Z-drugs | 97 (13.8) | 50 (13) | 147 (13.5) | 0.781 |
| **Distancing, first psychiatric exploration** | 340 (50.8) | 242 (65.9) | 582 (56.2) | <0.001 |
| Missing | 36 | 18 | 54 |  |
| **Distancing, second psychiatric exploration** | 254 (62.9) | 173 (76.2) | 427 (67.7) | <0.001 |
| Missing | 301 | 158 | 459 |  |
| **Follow-up therapy** |  |  |  |  |
| Outpatient psychiatric care | 15 (2.1) | 16 (4.2) | 31 (2.8) | <0.001 |
| Inpatient psychiatric care | 443 (62.8) | 166 (43.1) | 609 (55.9) |  |
| Discharge against medical advice | 168 (23.8) | 118 (30.6) | 286 (26.2) |  |
| Discharged home | 65 (9.2) | 71 (18.4) | 136 (12.5) |  |
| Other therapy | 8 (1.1) | 8 (2.1) | 16 (1.5) |  |
| Deceased | 6 (0.9) | 6 (1.6) | 12 (1.1) |  |

Data are n (%), unless otherwise indicated. Percentages may not total 100% due to rounding. OTC, over-the-counter medication; SRB, suicide-related behavior.
