# Supplementary material for: Socio-demographic and psychiatric profile of patients hospitalized due to self-poisoning with suicidal intention
Source: Ann Gen Psychiatry. 2022 Jun 9;21:16. doi: 10.1186/s12991-022-00393-3 (PMC9185897; doi:10.1186/s12991-022-00393-3)
Supplement: Supplementary file 4 — Additional file 4. Distribution of different psychiatric disorders among the patients classified into the combined psychiatric disorder group. [file 12991_2022_393_MOESM4_ESM.docx]

Additional file 4: Distribution of different psychiatric disorders among the patients classified into the combined psychiatric disorder group.
